# Effect of aliskiren on proteinuria in non-diabetic chronic kidney disease: a double-blind, crossover, randomised, controlled trial

**DOI:** 10.1007/s11255-011-0110-z

**Published:** 2012-01-28

**Authors:** Sławomir Lizakowski, Leszek Tylicki, Marcin Renke, Przemysław Rutkowski, Zbigniew Heleniak, Maja Sławińska-Morawska, Ewa Aleksandrowicz, Wieslawa Łysiak-Szydłowska, Bolesław Rutkowski

**Affiliations:** 1 Department of Nephrology, Transplantology and Internal Medicine, Medical University of Gdańsk. ul, Debinki 7, 80-211 Gdańsk, Poland; 2 Department of Clinical Nutrition and Laboratory Diagnostics, Medical University of Gdańsk, Gdańsk, Poland

**Keywords:** Aliskiren, Renin inhibitor, Proteinuria, Chronic kidney diseases, ACE inhibitor

## Abstract

**Aim:**

To evaluate the proteinuria-lowering effect of a renin inhibitor (aliskiren), compared to placebo and to an angiotensin-converting enzyme inhibitor (perindopril), in patients with non-diabetic chronic kidney disease.

**Methods:**

A randomised, double-blind, crossover trial was performed in 14 patients with nondiabetic chronic kidney disease with 24-h mean proteinuria of 2.01 g (95% CI, 1.36–2.66) and estimated creatinine clearance of 93 ± 6.8 ml/min. The study consisted of five treatment periods. The patients were randomly assigned to receive aliskiren (150 mg), aliskiren (300 mg), perindopril (5 mg), perindopril (10 mg) or placebo.

**Results:**

Aliskiren and perindopril reduced proteinuria. These effects were dose-dependent. Furthermore, 24-h proteinuria was reduced by 23% (mean 95% CI; 2–44) by treatment with aliskiren (150 mg), by 36% (95% CI, 17–55; *P* < 0.001) with aliskiren (300 mg), by 7.1% (95% CI, 11–26) with perindopril (5 mg) and by 25% (95% CI, 11–39; *P* < 0.05) with perindopril (10 mg), compared to placebo. No significant difference was found between the effects of aliskiren and perindopril.

**Conclusions:**

Aliskiren significantly reduced proteinuria. The antiproteinuric effect is probably similar to that of perindopril, for equivalent hypotensive dosages. The renin inhibitor provides a promising alternative approach for the treatment of patients with chronic proteinuric non-diabetic kidney disease.

## Introduction

Proteinuria is a major risk factor for the progression to end-stage renal disease in both diabetic and nondiabetic nephropathies [1]. Angiotensin II and aldosterone are the key players in the development of renal failure, acting directly to promote tissue fibrosis or indirectly on glomerular haemodynamics and proteinuria [2, 3]. Therefore, pharmacological inhibition of the renin–angiotensin–aldosterone system (RAAS) may have a beneficial impact on the progression of proteinuria and chronic kidney disease [3, 4].

Various studies have shown that treatment with angiotensin-converting enzyme inhibitors (ACEI) and angiotensin II receptor blockers (ARB) reduces both proteinuria and the rate of decline of the glomerular filtration rate in nondiabetic chronic renal disease [5–7]. Despite recent progress, however, there is still no optimal therapy that can stop the progression of these nephropathies. One reason may be the suboptimal suppression of RAAS activity via ACEI and ARB because a compensatory increase in renin concentration increases levels of angiotensin I and II. Angiotensin II can also be formed using pathways that do not involve the angiotensin-converting enzyme [3]. Therefore, it is necessary to search for alternative therapeutic strategies for blocking RAAS that can further improve renal outcome.

Recently, renin inhibitors, a new class of drugs that selectively inhibits angiotensin II formation at the first step of the RAAS cascade, have been introduced to clinical practice. Aliskiren is the first orally bioavailable direct renin inhibitor approved for the treatment of hypertension. The blood-pressure (BP)-lowering effect of aliskiren is associated with decreased synthesis of angiotensin I from angiotensinogen through inhibition of renin’s active enzymatic site [8].

Once-daily oral treatment with aliskiren lowers BP effectively in hypertensive patients, with a safety and tolerability profile comparable with that of a placebo [9, 10]. In some recent trials, aliskiren has also shown renoprotective potential in patients with type 2 diabetes and albuminuria [11, 12]. To date, however, limited studies have evaluated the renal effects of aliskiren in nondiabetic chronic renal diseases [13, 14]. Consequently, in the present study, we compared the short-term effects of treatment with aliskiren with those of the placebo and ACEI perindopril on proteinuria. In addition, we evaluated the tolerability of aliskiren and its effects on BP.

## Materials and methods

### Individuals

Patients were selected from the cohort that attended our renal outpatient department. The inclusion criteria were established as follows: age of 18–65 years, chronic nondiabetic proteinuric nephropathy (chronic kidney disease stage 1–3), stable proteinuria above 500 mg/24 h, blood pressure above 125/75 mm Hg and below 150/95 mm Hg and no steroids or other immunosuppressive treatment for a minimum of 6 months before the study. Patients with unstable coronary heart disease or decompensated congestive heart failure in the previous 6 months, patients with an episode of malignant hypertension or stroke in the history, patients with diabetes and patients with an estimated glomerular filtration rate of less than 30 ml per minute per 1.73 m^2^ of body-surface area were excluded. Stable proteinuria was defined as proteinuria with less than 20% variability during the 6 months preceding the study.

### General protocol

**Fig. 1:**
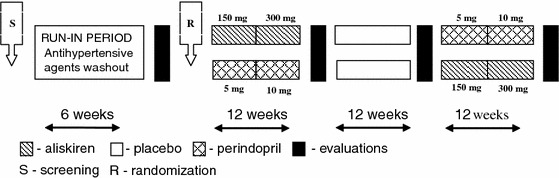
Scheme of the study

The study was a randomised, double-blind, controlled crossover trial in which the renal effects of therapy with aliskiren (A), perindopril (P) and placebo (PLACEBO) were compared. It consisted of a 6-week run-in period, 12 weeks of active treatment with aliskiren (Rasilez, Novartis) or perindopril (Prestarium, Servier) and 12 weeks of active treatment with the alternative medication after 12 weeks of placebo administration (Fig. 1). At the beginning of the study, the subjects who met the inclusion criteria began a 6-week run-in period during which the use of any previously used hypotensive agents was stopped. At the end of the run-in period, the patients were randomly allocated to one of the two treatment sequences: A/PLACEBO/P (sequence 1) or P/PLACEBO/A (sequence 2). The study medications were prepared, labelled and randomised by members of the staff at the Department of Pharmaceutical Technology, Medical University of Gdańsk. Allocation was performed by a person who was independent of the research team and according to a computer-generated randomisation list. For the first 6 weeks of the treatment period, aliskiren was used at a dose of 150 mg, and perindopril was administered at a dose of 5 mg. The dosages were doubled for the next 6 weeks to the maximal recommended hypotensive dosages of both study medications (i.e., aliskiren at 300 mg and perindopril at 10 mg). Increasing dosages of aliskiren (above 300 mg) and of perindopril (up to 12 or 16 mg) have been shown previously not to further reduce BP [9, 15]. Drug compliance was assessed by tablet count. The patients were instructed to take the study medication once daily in the morning. At the end of the run-in period, the administration of placebo, perindopril (5 mg), perindopril (10 mg), aliskiren (150 mg) and aliskiren (300 mg) was evaluated through measurements of 24-h ambulatory BP, 24-h proteinuria, serum creatinine and potassium levels. Estimated creatinine clearance was calculated as well. The patients were advised not to change their usual daily protein and sodium intake during the study period. The study was approved by the local ethical committee, and the investigated patients all provided their informed consent. The study was registered at http://www.clinicaltrial.gov (identifier: NCT01219413).

### Procedures and laboratory analyses

Ambulatory BP was measured continuously for 24 h using the Mobil-o-graph (version 12) monitoring system. BP was measured every 15 min during the day (7.00 a.m. to 10.00 p.m.) and every 30 min during the night (10.00 p.m. to 7.00 a.m.). The results of ambulatory BP measurements were analysed to determine mean 24-h systolic BP (SBP) and mean 24-h diastolic BP (DBP). Proteinuria and sodium (Na) and urea excretion were evaluated based on urine collection at 24 h. All of the patients were equipped with a scaled container and were strictly informed as to how to collect urine. Subjects collected two lots of urine at 24 h; using these samples, the mean value of 24-h proteinuria was calculated. The measurements of two samples collected within 1 week were averaged. The patients were asked not to perform heavy physical activity on the days of urine collection. Creatinine clearance was calculated according to the Cockroft-Gault equation.

### Statistics

Data from previous studies were used for the sample-size calculation. The primary end-point of this study was a difference in 24-h proteinuria samples from subjects treated with aliskiren compared with those given a placebo. The baseline 24-h proteinuria level was 2.0 ± 0.5 g/24 h. Assuming a 30% reduction in proteinuria after aliskiren treatment [11, 12], we predicted a decrease in proteinuria from 2.0 to 1.4 g/24 h with aliskiren therapy. Thirteen patients had to complete the study to give the study 80% power to consider differences as statistically significant (*P* < 0.05, 2-tailed) with an expected within-patient standard deviation of 0.5 g/24 h. The secondary aim was to compare the antiproteinuric effect of aliskiren and perindopril. Because of their skewed distributions, 24-h proteinuria and protein daily intake were logarithmically transformed before statistical analysis and expressed as geometric means and 95% confidence intervals. Other results are expressed as the mean ± SEM. The results from the end of the treatment period were compared with those at the end of the placebo period. Differences were assessed using the analysis of variance (ANOVA) for repeated measurements with Bonferroni corrections for paired comparisons. Subsequently, the effects of aliskiren (150 mg) were compared with the effects of perindopril (10 mg) using the *t*-test. A *P* value less than 0.05 (2-tailed) was considered statistically significant. The data were evaluated using the Statistica (version 7.1; StatSoft Inc., Tulsa, OK) software package.

To prevent or limit the possibility of a period effect, we introduced a degree of balance into the study design, with a scheme of randomisation that allowed every treatment to be represented in every period with the same frequency (Fig. 1). To prevent or limit the risk of a carryover effect, we planned each treatment period of 12 weeks. Previous studies showed that the effects of RAAS blockade on proteinuria are fully reversible within 4 weeks [16]. Thus, prolonging each treatment period for 12 weeks with alternating placebo periods allowed us to rule out a residual effect of previous treatment at the end of week 12 of another treatment, at which point proteinuria was measured. Grubbs’ test was used to detect outliers [17].

## Results

Of the 16 patients who entered the study, 14 (87.5%) completed the protocol. Two subjects dropped out because of the withdrawal of informed consent, which was not related to the side effects of the therapy. The baseline clinical characteristics of the patients who completed the protocol are listed in Table 1.

**Table 1:** Patients’ characteristic at baseline

Parameter	
*n*	14
Gender: female/male *n*	5/9
Age *years*	39.0 ± 3.94
Mean systolic blood pressure mm Hg	127 ± 3.4
Mean diastolic blood pressure mm Hg	79 ± 2.7
24-h proteinuria g	1,77 (1,36–2,66)
Serum creatinine mg/dl	0.96 ± 0.06
Creatinine clearance [Cockroft-Gault formula] ml/min	93 ± 6,8
24-hour urinary sodium mmol/24 h	219 ± 19
Serum potassium mmol/l	4.13 ± 0.38
Body mass index kg/m^2^	26.2 ± 1.0
*Diagnosis: n*	
Mesangial glomerulonephritis	3
Mesangiocapillary glomerulonephritis	1
Membranous glomerulonephritis	3
IgA nephropathy	1
Unknown nondiabetic proteinuric chronic kidney diseases	6
*Background hypotensive therapy: n*	
ACEI and ARB	7
ACEI (alone)	4
No hypotensive therapy	3

To convert serum creatinine in mg/dL to µmol/L, multiply by 88.4; eGFR in ml/min/1.73 m^2^ to ml/s/1.73 m^2^, multiply by 0.01667; data are expressed as mean ± SEM or geometric mean (95% CI)

### 24-h ambulatory BP

**Fig. 2:**
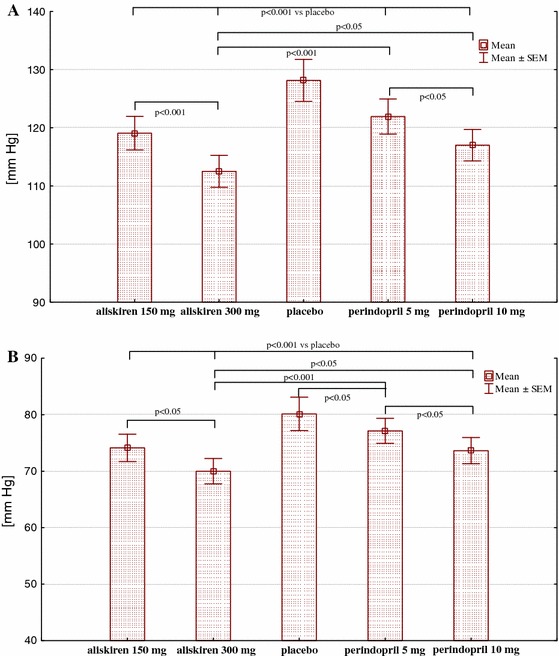
Systolic (**a**) and diastolic (**b**) blood pressure during study

SBP and DBP decreased significantly with aliskiren or perindopril treatments compared to those with placebo administration. Aliskiren (300 mg) was superior to perindopril (10 mg) for systolic (reduction of 15.6 mm Hg ± 1.6 vs. 11.1 ± 1.5; *P* < 0.05) and diastolic (reduction of 10.1 mm Hg ± 1.8 vs. 6.5 mm Hg ± 1.1; *P* < 0.05) (mean ± SEM) blood-pressure reduction (Table 2 and Fig. 2). Aliskiren (150 mg) and perindopril (10 mg) provided equal hypotensive efficacy.

**Table 2:** 24-h systemic blood pressure and laboratory results during study

	Placebo	Aliskiren		Perindopril	
		150 mg	300 mg	5 mg	10 mg
Systolic BP (24 h) mm Hg	128.1 ± 3.6	119.1 ± 2.8 ^a^	112.5 ± 2.7 ^adef^	121.9 ± 3.0 ^a^	117.0 ± 2.7 ^ab^
Diastolic BP (24 h) mm Hg	80.1 ± 2.9	74.1 ± 2.4 ^a^	70.0 ± 2.2 ^aefg^	77.1 ± 2.9 ^c^	73.62.2 ^ab^
24-h proteinuria g	1.89 (1.47–3.06)	1.25^c^ (0.96–2.55)	1.03 ^ab^ (0.76–1.96)	1.65 (1.24–3.02)	1.35 ^c^ (1.01–2.20)
Albumin/creatinine ratio (mg/mg)	0.74 ± 0.17	0.47 ± 0.1	0.34 ± 0.07^c^	0.48 ± 0.1	0.34 ± 0.07^c^
CrCl [CG] ml/min	92.4 ± 7.0	94.3 ± 7.4	91.6 ± 6.8	93.1 ± 5.8	94.26 ± 6.5
Serum creatinine mg/dl	0.99 ± 0.06	0.95 ± 0.06	0.98 ± 0.06	0.97 ± 0.05	0.95 ± 0.05
Serum potassium mmol/l	4.10 ± 0.06	4.22 ± 0.04	4.25 ± 0.09	4.05 ± 0.08	4.23 ± 0.06
Daily protein intake g/kg/24 h	0.85 (0.71–1.1)	0.86 (0.74–1.06)	0.90 (0.76–1.11)	0.89 (0.69–1.28)	0.93 (0.77–1.2)
Sodium urine excretion mmol/24 h	208 ± 23	202 ± 18	200 ± 24	180 ± 21	227 ± 29

Data are expressed as mean ± SEM or geometric mean (95% CI)

*BP* blood pressure; *CG* Cockroft-Gault formula

^a^Significant versus placebo (*P* < 0.001)

^b^Significant versus perindopril 5 mg (*P* < 0.05)

^c^Significant versus placebo (*P* < 0.05)

^d^Significant versus aliskiren 150 mg (*P* < 0.001)

^e^Significant versus perindopril 5 mg (*P* < 0.001)

^f^Significant versus perindopril 10 mg (*P* < 0.05)

^g^Significant versus aliskiren 150 mg (*P* < 0.05)

### 24-h proteinuria

**Fig. 3:**
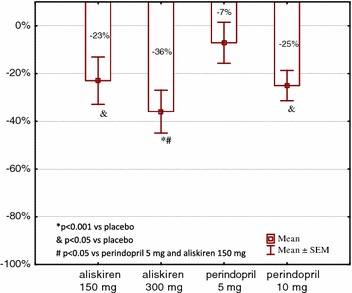
Changes in 24-h proteinuria versus placebo during study (mean ± SEM). *P < 0.001 versus placebo, *P* < 0.05 versus placebo, ^#^ *P* < 0.05 versus perindopril 5 mg and aliskiren 150 mg

Compared to the placebo values, 24-h proteinuria decreased by 23% (2–44, mean CI 95%) following aliskiren (150 mg) treatment, by 36% (17–55) following aliskiren (300 mg) treatment (*P* = 0.001), by 7.1% (11–26) following perindopril (5 mg) treatment and by 25.1% (11–39) following perindopril (10 mg) treatment (*P* = 0.04). In 9 of 14 patients, the maximal reduction in proteinuria was achieved with aliskiren (300 mg) and in another 5 subjects with perindopril (10 mg). The results showed that the reduction in 24-h proteinuria was comparable following equivalent hypotensive doses of both drugs (i.e., aliskiren at 150 mg and perindopril at 10 mg). In 7 of 14 patients, the reduction in proteinuria was greater with aliskiren at 150 mg. In the other 7 patients, the reduction in proteinuria was greater with perindopril at 10 mg (Table 2 and Fig. 3).

### Renal function, sodium and protein intake

Renal function as assessed by means of creatinine clearance remained stable during the study. There were no differences in sodium or protein intake between treatment periods (Table 2).

### Adverse effects: serum potassium concentration

Aliskiren and perindopril were well tolerated by the patients. Adverse effects were not reported. The serum potassium concentration was unchanged during the study period (Table 2).

## Discussion

In this exploratory short-term study, we demonstrated that treatment with a renin inhibitor, aliskiren, significantly reduced proteinuria in patients with nondiabetic chronic kidney diseases. Preclinical studies have shown that aliskiren, like other RAAS inhibitors, has antiproteinuric effects in both diabetic and nondiabetic models of chronic kidney disease. When it was compared with ACEI or ARB in these models, the renoprotective effects were approximately equal [18–20]. Clinical data on this point are still very limited and mainly focused on patients with diabetic nephropathy. In the AVOID trial, Parving et al. evaluated the effects of dual blockade of the RAAS with aliskiren and losartan in patients with hypertension and type 2 diabetes with nephropathy. Patients were maintained on losartan (100 mg daily) for the duration of the study and were randomised to receive a 6-month treatment with aliskiren or a placebo. After 3 months of treatment with aliskiren at 150 mg, albuminuria had been decreased by 11%. Increasing the dose of aliskiren to 300 mg caused a further decrease in the albuminuria to 20% of the baseline level [11]. In a double-blind, randomised, crossover study involving patients with type 2 diabetes, hypertension, and albuminuria, Persson et al. demonstrated that aliskiren treatment reduced albuminuria by 48% compared with a placebo. This reduction was not significantly different from the 58% reduction achieved with irbesartan treatment [21].

Studies concerning the above issue in patients with nondiabetic CKD are very limited. In two small studies, the addition of aliskiren to ARB was shown to decrease proteinuria in subjects with IgA nephropathy and various forms of primary glomerulonephritis [13, 14]. Aliskiren confers an antiproteinuric effect in patients who exhibit significant residual proteinuria despite having received the recommended renoprotective treatment. To our best knowledge, this is the first clinical study to perform a head-to-head comparison of renal effects between renin inhibitors and ACEI in patients with nondiabetic renal disease. Specifically, the study involved mainly patients with normal and high-normal systemic BP. Aliskiren was shown to reduce proteinuria compared to a placebo. The effect was dose dependent, as in the case of perindopril therapy.

In this study, we have shown that treatment with a renin inhibitor, aliskiren, significantly reduced proteinuria. In equivalent hypotensive doses, aliskiren seems to decrease proteinuria at least as efficiently as therapy with ACEI, perindopril. However, the study may have lacked sufficient power to identify small differences in the renal effects of these medications as significant. Increasing the dosage of aliskiren from 150 to 300 mg induced a further 13% decrease in protein excretion. Given the prognostic value of proteinuria for long-term renal outcome, it may be advisable to use aliskiren in therapeutic dosages that are as high as possible. This suggestion applies also to proteinuric patients with normal blood pressure. The therapy with aliskiren even in maximal dosages of 300 mg was well tolerated by all patients. Systolic blood pressure did not decrease below 110 mm Hg. Of particular interest may be the renal effects of aliskiren at levels that exceed maximal recommended dosages.

Aliskiren was shown to be a more potent hypotensive drug than perindopril. At the maximal registered doses, aliskiren at 300 mg was superior to perindopril at 10 mg for the reduction in systolic and diastolic BP. This finding is in line with comparative studies showing that aliskiren may be slightly more effective in lowering BP than ACEI [22, 23]. A greater reduction in BP leads to greater proteinuria reduction. In our study, aliskiren at 300 mg reduced proteinuria to a greater extent than perindopril at 10 mg; however, the sample size of the study was too small to identify this trend as significant. One may not exclude, however, that this effect was also the consequence of more comprehensive suppression of RAAS components during aliskiren treatment. Direct renin inhibitors may provide more complete and thus more effective blockade of the RAAS than treatment with ACEI or ARB. Fisher et al. [24] showed that maximal doses of aliskiren increased renal blood flow to levels twofold greater than those achieved by maximal doses of ACEI, captopril; the effect was 40% stronger than that achieved by ARB.

It is unlikely that other confounders influenced the study outcome. The five treatment periods did not differ with respect to renal function or protein intake. To reduce the influence of sodium-dependent mechanisms on the study results, no diuretics were allowed during the study. In addition, patients were instructed not to change their daily sodium intake during the study period. Sodium excretion was monitored and found not to change during all study phases. Our exploratory study had enough power to detect a significant difference in antiproteinuric effect between aliskiren and placebo but lacked sufficient power to detect such a difference between both dosages of each medication. A potential limitation may also be the fact that patients with severe nephrotic syndrome or impaired renal function were not evaluated. Furthermore, a comparable reduction in proteinuria over 6 weeks may not necessarily suggest that aliskiren has a similar long-term effect to an ACEI on the rate of GFR decline.

Aliskiren therapy was well tolerated by patients with no incidence of hyperkalaemia or acute renal failure. There were also no differences between aliskiren and perindopril with respect to changes in serum potassium or renal function.

In conclusion, aliskiren significantly reduced proteinuria in a dose-dependent manner. This effect is probably at least as effective as perindopril in equal hypotensive doses. Given the strong prognostic value of proteinuria for evaluating renal outcome, aliskiren provides a promising alternative approach for the treatment of patients with nondiabetic chronic kidney diseases, even in those without hypertension.
